# Priapism as the initial symptom of primary penile lymphoma: A case report

**DOI:** 10.3892/ol.2014.2488

**Published:** 2014-08-28

**Authors:** ZHAOHUA GONG, YING ZHANG, HONGJIN CHU, PEIWEN LIAN, LIANGMING ZHANG, PING SUN, JIAN CHEN

**Affiliations:** 1 Department of Oncology, Yantai Yuhuangding Hospital Affiliated to Qingdao University, Yantai, Shandong 264000, P.R. China; 2 Division of Graduate Education, Dalian Medical University, Dalian, Liaoning 116027, P.R. China; 3 Central Laboratory, Yantai Yuhuangding Hospital Affiliated to Qingdao University, Yantai, Shandong 264000, P.R. China

**Keywords:** priapism, primary penile lymphoma, metastasis

## Abstract

Primary penile lymphoma presenting with priapism as the initial symptom is extremely rare. In total, <10 cases have been previously reported. The diagnosis can be difficult and patients often develop metastasis. The current study reports the case of a 48-year-old male, who presented with a one-month history of painless priapism. On admission to Yantai Yuhuangding Hospital Affiliated to Qingdao University (Yantai, China), examination revealed an erect penis, enlarged lymph nodes in the bilateral inguinal and swelling in the thighs. A biopsy was taken from the right inguinal lymph node and the pathological diagnosis confirmed a diffuse large B-cell type of non-Hodgkin’s lymphoma, while an enhanced computed tomography scan of the chest revealed evidence of the invasion of malignant lymphoma cells. Priapism disappeared two days following the completion of the first cycle of chemotherapy with the E-CHOP regimen (cyclophosphamide, vincristine, prednisone, epirubicin and etoposide); however, evidence of brain metastases was observed one month later, which was confirmed by magnetic resonance imaging. The patient received cranial radiotheraphy and systemic treatment for cerebral edema. The patient did not respond well to treatment and succumbed to the disease three months following the initial diagnosis of lymphoma. Lymphoma may be difficult to diagnose, depending on the initial symptoms; therefore, the patient history must be carefully assessed so as to determine an early diagnosis and prevent metastasis, thus improving the prognostic outcome.

## Introduction

Primary penile lymphoma presenting with priapism as the initial symptom is extremely rare, with few cases reported worldwide. Penile cancer accounts for only 0.4–0.6% of all malignancies in the developed world (1), and the involvement of lymphoma is even more rare. The first case of primary malignant lymphoma of the penis was reported in 1962 (2), and few cases have been reported since. The most common subtype is diffuse large B-cell lymphoma (3). The diagnosis of rare lymphoma is difficult and may be delayed without specific symptoms and thus, treatment of this lesion remains controversial. Chemotherapy is the main treatment adminstered, however, immunotherapy, radiotherapy and penile preservation have all been reported (3,4) and a combined treatment modality is usually recommended (5). The current study reports a patient diagnosed with primary malignant penile lymphoma, with metastasis in the lungs and brain, and poor prognosis. The diagnosis was confirmed by biopsy, and enhanced CT of chest revealed evidence of pulmonary and brain metastasis. Written informed consent was obtained from the patient.

## Case report

**Figure 1:**
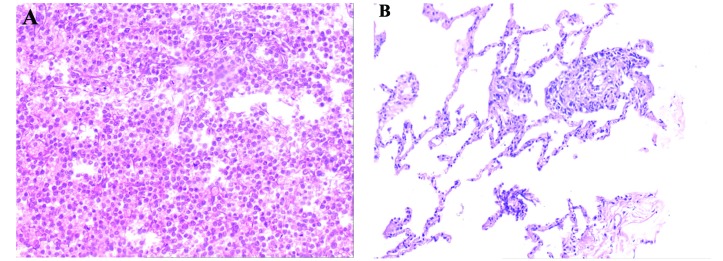
Biopsy taken from the right inguinal lymph node showing (A) diffused infiltration of atypical neoplastic lymphocytes with primitive nuclear morphology, visible nucleoli and little cytoplasm. The cells exhibited positive immunoreactivity for CD20. (B) Biopsy of the lung revealed diffused infiltration of atypical neoplastic lymphocytes by hematoxylin and eosin staining. Original magnification, ×200.

A 48-year-old male presented to Yantai Yuhuanding Hospital Affiliated to Qingdao University (Yantai, China) with a one-month history of painless priapism associated with chest congestion and shortness of breath. The patient had a history of type II diabetes. Physical examination revealed a swollen penis, enlarged inguinal lymph nodes, swelling in the thighs and varicose veins, which were visible in the lower limbs. No fever, night sweats or weight loss were observed in the month prior to patient referral, and no symptoms of ulceration, difficulty in urination or complaint of any epidermal changes were observed; therefore, no systemic symptoms of lymphoma were presented. Norepinephrine was administered, however, detumescence did not occur. The B-mode ultrasound examination of the rectum and perineum and the arteriography revealed no apparent arteriovenous fistula. An abdominal and pelvic computed tomography (CT) scan showed multiple node involvement, including the aortoiliac vessels, neck of the bladder, seminal vesicle, prostate gland and bilateral inguinal area. A biopsy was taken from the right inguinal lymph node (1.5×1.5 cm) and immunohistochemical analysis with markers confirmed that the lymphoma cells were CD20^++^, CD3^−^, CD10^−^, MuM1^−^ and bcl-6^−^, corresponding with non-Hodgkin’s lymphoma (Fig. 1). The degree of staining was calculated using the following scale: −, positive staining of cancerous cells was observed in <25% of the cells; +, positive staining of cancerous cells was observed in >25% but <50% of the cells; ++, positive staining of the cancerous cells was observed in >50% but <75% of the cells (6). Based on these observations, the diagnosis of diffuse large B-cell lymphoma (DLBCL) was determined.

**Figure 2:**
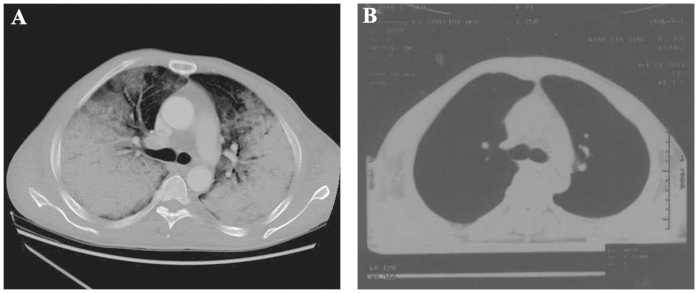
Transaxial computed tomography images of the chest showing infiltration of the lungs (A) prior to chemotherapy. The bilateral lungs had a large area with a dark appearance, and mediastinal lymph nodes were visible.(B) Transaxial computed tomography images of the chest following chemotherapy.

One week following admission, the patient complained of chest congestion; an enhanced CT scan revealed the invasion of malignant lymphoma cells to the lungs, and pericardial and bilateral pleural effusion was also identified (Fig. 2); however, at this stage, the possibility of fungal infection was not excluded. The E-CHOP regimen was administered (1.2 mg cyclophosphamide, day one; 2 mg vincristine, 60 mg epirubicin, days one to two; 100 mg prednisolone, days one to five; and 0.1 mg etoposide, days one to five). After four days, chest congestion, edema in the limbs and priapism were moderately relieved. A further histology of the lung by a needle biopsy revealed lymphoma cell infusion. Treatment for the decreased white blood cell count (0.56×10^9^ cells/l; normal range, 4–10×10^9^ cells/l) was administered during the second course of chemotherapy. The patient was continuously treated with systemic chemotherapy with the E-CHOP regimen. Concomitantly, symptomatic treatment to relieve airway spasms was administered. The patient tolerated the entire course of chemotherapy well, and the priapism was alleviated two days following the completion of one cycle of E-CHOP therapy. The enlarged bilateral inguinal lymph nodes were non-palpable at the time of completion.

**Figure 3:**
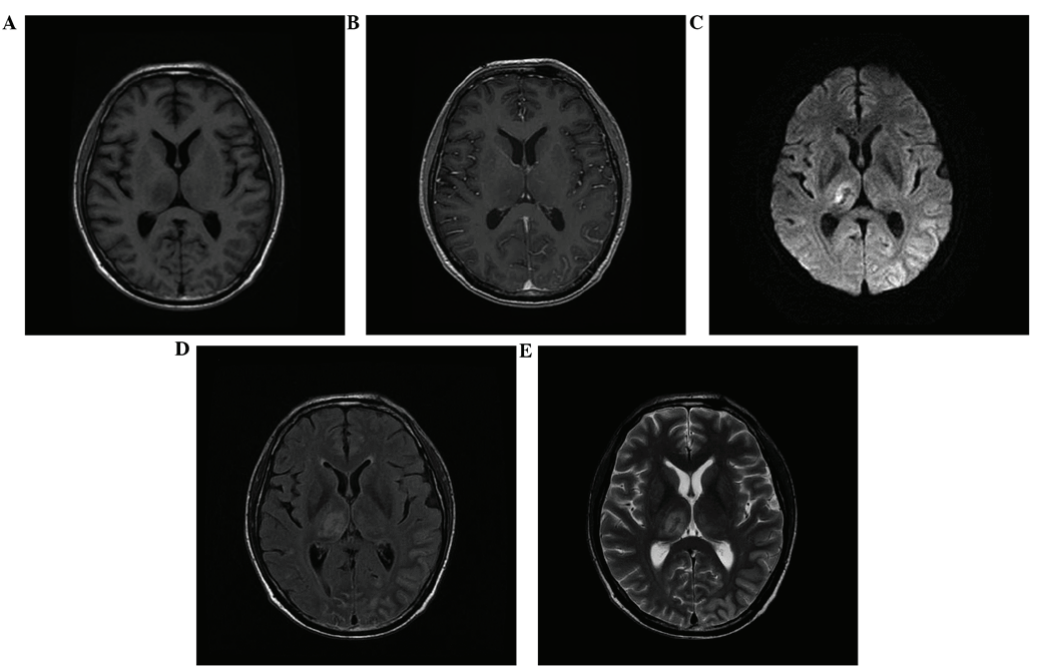
Magnetic resonance imaging scans show cranial nerve nuclei involvement of lymphomatic metastasis. The bilateral cerebral hemisphere cortex had (A and B) marginally long T1 and (C) long T2 signal changes, and cerebral fissure was not apparent. Bilateral basal ganglia had hyperintense T1 and T2 signals. (D) Fluid attenuated inversion recovery showed enhanced signal, whereas (E) diffusion weighted imaging exhibited a slightly low signal on the right. No apparent enhancement of the signal was observed following the injection of the contrast agent.

On day 14 following admission, the patient was unresponsive to external stimuli and suffered an epileptic seizure. The results of the laboratory evaluations were as follows: Blood ammonia levels, 18 μmol/l (normal range, 20–60 μmol/l); blood ketone bodies, negative; and blood sugar levels, 17.27 mmol/l (normal range, 3.9–7.5 mmol/l); therefore, DKA and hepatic encephalopathy were ruled out. A cranio-cerebral magnetic resonance imaging scan revealed an anomaly in the rear of the anterior and posterior pituitary, suggesting possible cranial nerve nuclei involvement and infiltration (Fig. 3). The patient was subjected to radiotherapy when brain metastasis was identified. Systemic treatment for cerebral edema (dehydration) and nutrition following severe epileptic seizures were also administered. The final diagnosis was stage IV diffuse large B-cell non-Hodgkin’s lymphoma with metastasis to the brain and lungs, according to the Ann Arbor-Cotswolds staging classification (7). The patient succumbed to the disease three months following the initial diagnosis of lymphoma.

## Discussion

Priapism is defined as an involuntary, usually painful, prolonged penile erection unrelated to sexual stimulation (8). It is most commonly caused by hematological disorders such as sickle cell anemia; however, other causes include neurological damage, trauma, infection, malignancy, erectile dysfunction drugs, such as papaverine and alprostadil, and metabolic disturbances (9). The presentation of penile lymphoma varies, with symptoms including indurated papules, nodules, ulcers and diffuse penile swelling (10). Primary penile lymphoma is extremely rare and difficult to diagnose, and patient history must be carefully assessed. In order to determine an accurate diagnosis, a full physical examination, radiological imaging studies, excision biopsy and immunohistochemical analysis must be conducted. Reports have been published describing this malignancy with common phenotypes including nodules, ulceration or penile enlargement, all of which are similar to other soft tissue tumors and, therefore, lead to the condition being difficult to diagnose (3). However, priapism as the initial symptom of lymphoma is extremely rare. To the best of our knowledge, only four cases of primary penile lymphoma have been reported presenting with priapism as the initial symptom (11–13). The pathological studies demonstrated that all cases were diffuse large DLBCL, which is the most common subtype of lymphoma at this anatomical site (14).

In the current study, the 48-year-old male initially presented with priapism, which was followed by enlargement of the lymph node. The pathological studies confirmed the diagnosis of malignant penile lymphoma and treatment with the E-CHOP regimen led to the remission of the physiological symptoms; however, the patient later developed metastasis in the lungs and brain. This indicated a poor prognosis for DLBCL, the most common subtype of non-Hodgkin’s lymphoma. DLBCL is aggressive and is associated with a wide range of clinical manifestations at all ages; however, patients with the condition may undergo complete remission with appropriate treatment (11,12,15,16). It was speculated that priapism in lymphoma is caused by tumor cells infiltrating the penile cavernous tissue, which causes venous obstruction and priapism (10). Priapism caused by tumor cell infiltration is usually associated with congestion and swelling in the body of penis. Additionally, priapism as the initial manifestation has been reported in leukemia, which is also a malignant hematological disease. Often, priapism subsides after two to seven days of chemotherapy; however, occasionally it is caused by tumor lysis syndrome in leukemia, where the condition occurs following chemotherapy (13,17). To achieve an improved outcome in cases where the original diagnostic evidence is limited or current treatment failed, the possibility of lymphoma must be investigated. Furthermore, it has been suggested that levels of soluble interleukin-2 receptor may serve as a potential prognostic marker for the low complete response rates in DLCBL (16,18).

To date, the preferred treatment for DLCBL includes excision, radiotherapy, and chemotherapy; however, no standard treatment modality has been established (16). Although early-stage localized lymphoma is potentially curable with localized therapy and has a good prognosis, the anatomical resection and radiotherapy may cause local morbidity. The E-CHOP regimen alone, or combined with rituximab, which is a chimeric monoclonal antibody against the CD20 B-cell antigen, has become the current recommended treatment for DLBCL. In the majority of reported cases, following chemotherapy treatment, complete resolution of the disease was observed for between four months and six years (10,15,19–26). However mortalities due to this malignancy have also been reported (21).

In conclusion, the current study reports the case of a 48-year-old male with primary penile lymphoma, presenting with a one-month history of priapism due to large DLBCL cell infiltration, which also affected the lungs and cranial nervous system. The diagnosis was prolonged as the initial manifestation of the disease was extremely rare. The E-CHOP chemotherapy regimen was the selected treatment modality in this case, considering the patient’s relatively young age, due to the vigorous nature of the therapy. However, the prognosis was poor due to the advanced stage of the invasion and disseminated lymphoma cells. This case report may increase understanding with regard to the specific aetiology and pathogenesis of this disease and may prevent misdiagnosis by clinicians. Non-Hodgkin’s lymphoma may be difficult to diagnose based on the initial symptoms presented by patients. Thus, early recognition and appropriate clinical management are required. Further clinical studies are required to identify a standard treatment for this malignancy.
